# Utilization of Mind–Body Intervention for Integrative Health Care of COVID-19 Patients and Survivors

**DOI:** 10.3390/ijerph19116618

**Published:** 2022-05-29

**Authors:** Hyun-Jeong Yang, Noriko Setou, Eugene Koh

**Affiliations:** 1 Korea Institute of Brain Science, Seoul 06022, Korea; 2 Department of Integrative Health Care, University of Brain Education, Cheonan 31228, Korea; 3 Department of Disaster Psychiatry, Fukushima Medical University, Fukushima 960-1295, Japan; setou@fmu.ac.jp; 4 Temasek Life Sciences Laboratories, Singapore 117604, Singapore; eugene@tll.org.sg

**Keywords:** Mind–Body Intervention, diabetes, immune, COVID-19, long COVID, stress, mental health, blood glucose

## Abstract

Recent findings suggest a correlation between COVID-19 and diabetes, although the underlying causes are still little understood. COVID-19 infection tends to induce severe symptoms in patients with underlying diabetes, increasing their mortality rate. Moreover, COVID-19 itself appears to be a diabetogenic factor. In addition, mental health conditions, such as depression due to lockdown and anxiety about infection, were found to affect glycemic control and immunity, highlighting the importance of mental health care during the pandemic. Mind–Body Intervention (MBI), which includes meditation, yoga, and qigong, has emerged as a tool for mental health management due to its effects on stress reduction and the promotion of mental and physical well-being. Here, we review the latest randomized controlled trials to determine the effects of MBI on glycemic control and the immune system and discuss the underlying mechanisms by which MBI facilitates the virtuous cycle of stress management, glycemic control, and immune modulation. Furthermore, we examine the actual utilization of MBI during the COVID-19 pandemic era through recent studies. With proper online education, non-pharmacological MBI may be more widely used as an important tool for self-health care that complements the usual treatment of COVID-19 patients and survivors.

## 1. Relationship between COVID-19 and Diabetes, and the Potential Benefits of Mind–Body Intervention (MBI)

Patients with pre-existing conditions including diabetes may have a worse prognosis from coronavirus disease 2019 (COVID-19) infections. During the pandemic, lockdown-mediated social isolation, uncertainty, anxiety and reduced physical activity can aggravate diabetes-specific emotional distress, which may decrease both compliance to therapeutic regimens and glycemic control. Poor mental health and socioeconomic issues caused by COVID-19 or post-COVID may also increase the risk of type 2 diabetes mellitus (T2DM). Non-pharmacological therapeutic approaches such as Mind–Body Intervention (MBI), although mainly used as a stress reduction strategy, were also reported for their modulating effects on both glycemic control and immune function, suggesting their potential use in self-health care during the pandemic. Given the relatively recent occurrence of COVID-19, not many studies have examined the application of MBIs for the prevention or recovery of COVID-19. Here, we approach this review by systematically examining the effects of MBI on factors correlated with disease severity and mortality from COVID-19 such as glycemic state and immunological factors. Following this, we explore studies that examined the effects of MBI on depression, anxiety, and stress associated with poor immune function, which can aggravate negative clinical outcomes of COVID-19. Lastly, we discuss current studies that have examined the effects of COVID-19 on the physical and mental health of COVID-19 patients, as well as caregivers. We hope that this study can provide a basis for the practical use of MBI not only for the management of mental health, but also in glycemic control and immunity during the pandemic.

Recent studies indicated a possible bidirectional relationship between COVID-19 and diabetes. Diabetes patients have an increased risk of exhibiting severe COVID-19 clinical outcomes, that is, higher rates of hospitalization and mortality [1]. Moreover, new-onset diabetes and severe metabolic complications of pre-existing diabetes (including ketoacidosis and hyperosmolarity) have often been observed in patients with COVID-19 [2,3,4]. Severe acute respiratory syndrome coronavirus 2 (SARS-CoV-2), which causes COVID-19, binds to angiotensin-converting enzyme 2 (ACE2) receptors expressed in major metabolic tissues, including pancreatic beta cells, adipose tissue, small intestine, and kidneys [5]. These events induce a variety of changes in glucose metabolism, which complicates the pathophysiology of pre-existing diabetes or triggers novel mechanisms in the disease. Other coronaviruses, which bind to ACE2 receptors, often induce ketosis-prone diabetes [6]. There were higher incidences of fasting glycemia or acute-onset diabetes in SARS coronavirus 1 pneumonia patients than in non-SARS pneumonia patients [6].

As COVID-19 continues to spread globally, leading to a substantial number of confirmed cases, health care crises have emerged in several cities worldwide, causing an increase in individuals needing home care for COVID-19 infection and other pre-existing diseases. During this pandemic, public education regarding non-pharmacological MBI techniques complementary to regular therapy would help self-care and health promotion for affected individuals and the general public. This paper is a comprehensive review of recent studies on the effects of MBIs on glycemic control, the immune system and mental health, together with their potential underlying mechanisms. Moreover, it includes recent studies regarding the application of MBI in COVID-19 patients/survivors and health care providers, patients with other diseases, and healthy populations during the current pandemic.

MBIs (also called mind–body therapies, mind–body training, mind–body medicine) include a wide range of strategies such as meditation, yoga, tai chi, and qigong. Many of these strategies often emphasize focusing on the present moment, resulting in stress reduction, mental health improvements, and beneficial effects on physical health [7,8,9,10]. As one’s ability of awareness, either of one’s own body senses or the outside world, can be mainly cultivated through various traditions of MBI practices, the effects and mechanisms of MBIs might be fundamentally different from general exercise or resting behavior. Studies have shown that MBIs exert hypoglycemic [11,12,13,14,15,16], as well as immune-modulating effects [17,18], implying their potential contribution toward improving the prognosis of COVID-19, a potentially diabetogenic infectious disease.

## 2. Literature Review Methods and Findings

### 2.1. Search Strategy

Although this study is not a systematic review, in order to provide transparency in the search and selection process, we followed the guidelines of the Preferred Reporting Items for the Systematic Reviews and Meta-Analysis (PRISMA) statement [19]. PubMed was searched for articles published within the last 5 years (March 2017~May 2022) regarding the effects of MBI (keywords: yoga, qigong, tai chi, meditation, MBSR, Mind–Body Intervention, mindfulness) on glucose-related biochemical measures (keywords: glucose, HbA1c, insulin) or on immune function-related measures (keywords: CRP, interleukin, TNF, interferon, B cells, NK cells, T cells) by all combinations of two keywords consisting of an intervention and an outcome (e.g., “yoga, HbA1c”) in titles and abstracts.

### 2.2. Selection Criteria

Articles were eligible if they were (1) randomized controlled trials (RCTs), (2) published in English, (3) published within the last 5 years. Moreover, they were included if they reported (4) a Mind–Body Intervention as a main intervention, (5) glucose-related biochemical measures as research outcomes, and (6) immune function-related measures as research outcomes. Studies were ineligible if they were (1) case reports, protocol papers, reviews or theoretical articles; (2) book chapters; and (3) studies whose main interventions were not MBIs. In addition, studies were excluded (4) if the study outcomes were not related to glycemic or immune function, (5) if the number of participants in the study was fewer than 20, and (6) if studies were retracted. Articles were screened for eligibility by specific inclusion and exclusion criteria. From the selected articles, information was extracted by assessing the full text.

By the above mentioned search methods, 176 papers were identified from Pubmed and 95 duplicate papers were removed. Among 81 papers assessed for eligibility, the following number of papers was excluded with indicated reasons: diet intervention (*n* = 13); acupuncture/accupressure intervention (*n* = 3); protocols (*n* = 3); lifestyle intervention (*n* = 1); no MBI interventions (*n* = 8); outcomes not related to glycemic or immune function (*n* = 6); nutrients intervention (*n* = 2); drug intervention (*n* = 1); retracted (*n* = 1); reviews (*n* = 2) (Supplementary Figure S1).

### 2.3. Study Quality Assessment

To assess study quality, checklists of the National Heart, Lung, and Blood Institute (NHLBI) were used [20] with minor modifications by omitting items related to blinding due to their impracticality in MBI studies [18,21] (Supplementary Table S1). The followings are descriptions of each item for quality assessment: 1. RCT; 2. adequate randomization method; 3. similarity of groups at baseline; 4. drop-out rate less than 20% at end point; 5. differential drop-out rate less than 15%; 6. adherence to intervention protocols; 7. similar background intervention; 8. valid and reliable outcome measurement; 9. power calculation; 10. pre-specified outcomes; 11. intention-to-treat analysis. Items were evaluated as ‘yes (present)’ or ‘no (absent)’; one point or zero points were added according to the evaluation, respectively. Total score was 11 for the highest quality paper. Study quality was considered higher if the score was close to 11. The 41 papers that were analyzed received the following assessments: score 10 (9 articles); score 9 (14 articles); score 8 (12 articles); score 7 (5 articles); score 6 (1 article).

## 3. Potential Effects of MBI on Glycemic Control

A number of studies reported the effects of MBIs on blood glucose, glycated hemoglobin levels, and insulin resistance in a variety of people, including those with T2DM, metabolic syndrome, overweight/obesity, or cardiovascular risk, some of which reported beneficial outcomes (Table 1).

One study showed that 3 months of yoga intervention lowered blood glucose and glycated hemoglobin (HbA1c) levels in T2DM patients, whereas standard walking exercises had no such effects (*n* = 48) [22]. However, another RCT for patients with T2DM (*n* = 87) showed that 3 months of qigong or tai chi did not induce significant changes compared to stretching (control group) [23]. For type 1 diabetes patients, 9 weeks of diabetic support significantly reduced HbA1c, whereas mindfulness-based stress reduction (MBSR) or cognitive–behavioral stress management did not (*n* = 48) [24]. The frequency of training sessions might also contribute to positive outcomes as an increased number of training sessions per week resulted in decreased glucose levels [22] while a single training session per week did not [23,24] if home practices were not counted, in T2DM patients.

Several studies have reported on the application of MBIs on blood sugar management among people with metabolic syndrome. Tai chi significantly reduced fasting blood glucose (FBG) and HbA1c levels compared to brisk walking and usual activity in this population (*n* = 246) [25]. In another study, yoga intervention was combined with a dietary regime. In this study, no significant differences were observed in fasting plasma glucose (FPG) levels between the yoga-based intervention group and the diet-only intervention group (*n* = 260) [26]. The impact of diet might be stronger than the MBI in the glycemic control, as there were no additional effects found in MBI plus diet compared to the diet-only in FPG level [26]. Additionally, in another study, three months of tai chi promoted no change in glycemic management (*n* = 54) [27]. Considering the long-term (6 and 9 months) effects of tai chi on glycemic control [25], changes might be observed after a longer period of MBI practice.

MBIs have been reported to be beneficial in lowering blood glucose levels of other susceptible populations, such as people with pre-diabetes, obesity, family history of T2DM, and high risk of ischemic stroke. An RCT involving women with pre-diabetes showed that 3 months of a diabetic yoga protocol significantly reduced the level of HbA1c and FPG levels compared to the waitlist control (*n* = 37) [28]. In women with body mass index ≥ 25, 8 weeks of MBSR significantly decreased FPG levels 8 weeks after the intervention and upon follow-up assessment after 16 weeks compared to health education (control) (*n* = 86) [29]. Meditation seems to be effective for relieving concerns regarding diabetes among not only adults but also adolescents. Adolescent girls with overweight/obesity, family history of diabetes, and elevated depressive symptoms were randomized into mindfulness-based group intervention or cognitive–behavioral intervention and subjected to 6 weeks of practice. Compared to the cognitive–behavioral intervention, the mindfulness-based group intervention showed significant reductions in fasting insulin levels and insulin resistance 6 weeks after the intervention but not at the follow-up assessment after 6 months. However, FBG was not altered at either 6 weeks or 6 months in both groups (*n* = 33) [30]. Individuals with cardiovascular diseases, including stroke, are recommended to engage in physical activity [36], which has been associated with the incidence of stroke in adults [37]. An RCT involving older adults at high risk for ischemic stroke revealed that 12 weeks of tai chi training significantly reduced FBG levels compared to the usual physical activity (control group) (*n* = 170) [31], suggesting that tai chi training provides sufficient improvements in glycemic control of elderly people at high risk for ischemic stroke. Genetic predisposition has been considered one of the major risk factors for diabetes [38,39,40,41,42]. In fact, an RCT on non-diabetic offspring whose parents have diabetes found that 8 weeks of yoga significantly reduced FPG levels, oral glucose tolerance test post-2 h glucose levels, fasting insulin levels, and insulin resistance, whereas the control group showed no significant changes (*n* = 57) [32]. This implies that yoga practice can effectively relieve the aforementioned pre-diabetes features. The aging process increases cellular stress and damage in pancreatic β cells, thereby reducing the insulin secretion capacity of β cells and increasing insulin resistance, leading to dysregulation of glucose control and age-related diabetes [43]. Therefore, glycemic control is critical for older adults. Among elderly populations, 9 weeks of mindfulness program significantly reduced HbA1c levels, whereas routine care promoted no significant changes (*n* = 120) [33], suggesting the beneficial effects of MBI on glycemic control among the elderly. In the above mentioned susceptible populations without disease, MBI was relatively effective in glycemic modulation.

However, MBI was not effective for glucose regulation of cardiac patients under cardiac rehabilitation. In an RCT involving patients undergoing cardiac rehabilitation following acute coronary events, 3 months of yoga practice showed no difference in FBG compared to the usual care (*n* = 60) [34]. In another study for cardiac patients, no change was found in HbA1c level after 8 weeks of MBSR program (*n* = 47) [35].

Although many studies have reported the beneficial effects of MBI on glycemic regulation, variations in its effects have been noted perhaps due to the different health states of subject populations, their education level, and the intensity, frequency, and period of MBI performance, warranting further studies to understand which conditions most effectively regulate glucose level to improve the use MBI for self-care.

## 4. Potential Effects of MBI on the Immune System

Glycemic regulation interacts with the innate immune system. Diabetes is accompanied by systemic low-grade inflammation, and chronic activation of the innate immune system dysregulates insulin secretion and contributes to diabetes complications [44]. MBI has also been studied for its effects on immune function; inflammation, and cell-mediated immunity [17,45]. Studies performed in wide range of populations, including healthy individuals, lonely elderly, people with depression or anxiety disorders, cancer patients, rheumatoid arthritis patients, those with metabolic syndrome, myocardial infarction, mild cognitive disorders, fibromyalgia, glaucoma, human immunodeficiency virus (HIV)-1, parkinson’s disease (PD) and cardiac rehabilitation, have implied the potential impacts of MBI on immune function (Table 2).

During infection, pro-inflammatory markers—C-reactive protein (CRP), interleukin-6 (IL-6) as well as interferon-gamma-inducible protein-10 (IP-10)—respond acutely. In chronic inflammatory conditions, such as diabetes, systemic levels of these markers remained increased for an extended period of time [73,74]. Moreover, inflammation also increases in the aging process [75]. In some healthy populations, MBI was found to have impacts on inflammatory marker expression and modulating immune cells. In an RCT, 413 healthy middle-aged adults were randomized into three groups: MBSR, aerobic exercise, or waitlist group [46]. Time had a major effect on CRP within the MBSR group; with a reduction in CRP level after 17 weeks but not 8 weeks. Other inflammatory markers, including IL-6 and IP-10, were not significantly altered following MBSR [46]. Aerobic exercise did not change CRP or IL-6 but significantly reduced IP-10 levels after the intervention (8 weeks) and upon follow-up (17 weeks), whereas no changes in the waitlist group were observed at all time points. This result implies a distinct working mechanism between MBI and physical exercise in middle-aged adults. Similarly, CRP levels were not changed in another RCT right after 8 weeks of MBSR [47], suggesting that a longer period of MBSR training may be required to observe these changes. In younger adults, MBSR exhibited a reduction in the secretion of one inflammatory marker secretion (IL-8), with no significant changes in other inflammatory markers [48]. In adolescents, MBSR was not effective in immune modulation, with no changes in the levels of CRP or IL-6 [49]. In another RCT among healthy adults, 3 months of qigong training significantly reduced peripheral IL-6 levels (*n* = 48) [50]. In addition, 3 months of contemplative mental training cultivating interception and present-moment focus (presence module) significantly reduced IL-6 and CRP in people with a high inflammatory load [51].

For healthy people, acute psychological stress increases circulating levels of peripheral blood natural killer (NK) cells, which are major constituents of the innate immune system, implying a potential for the development of allergic and autoimmune disorders [76]. On the other hand, chronic stress-induced glucocorticoids can cause adaptive immune deficiency via the apoptosis of B and T lymphocytes in mice [77]. An RCT involving 43 healthy people found that 1 month of qigong practice significantly reduced the percentage of NK cells and increased the number and the percentage of B lymphocytes, which are key components of adaptive immunity [52]. This indicated that short-term MBI could exert an immunomodulatory action on innate and adaptive immune responses. However, some studies found that MBI had no immune-regulating effects. In an RCT involving 54 healthy university students, 8 weeks of mindfulness training did not induce any changes in the participants’ immune systems (no changes in the levels of cortisol, CRP, IL-8, tumor necrosis factor-alpha (TNF-α), %CD4^+^ T lymphocytes, %CD8^+^ T lymphocytes, %CD19^+^ B lymphocytes, %CD14^+^ monocytes, and %NK cells) [53], implying that MBI had different effects depending on the population demographic.

Mental conditions such as depression and anxiety are strongly linked to low-grade systemic inflammation [78]. In RCTs for patients with major depression [54] or anxiety disorder [55], MBIs significantly reduced inflammatory cytokines including IL-6, suggesting the potential use of MBIs to improve depression- or anxiety-related low-grade inflammation. Low-grade inflammation was also observed in conditions of neurodegenerative disorders. A meta-analysis of 170 studies showed that elevated inflammation is a feature of mild cognitive impairment and Alzheimer’s disease [79]. Compared with the control, in the periphery, higher levels of soluble TNF receptor2 (sTNFR2), IL-6, monocyte chemoattractant protein-1 (MCP-1) were identified in MCI, and levels of high-sensitivity CRP (hs-CRP), IL-6, sTNFR1, sTNFR2, IL-1β increased in AD. In patients diagnosed with MCI, 8 weeks of MBSR in comparison to cognitive training significantly reduced the activation of some subsets of monocytes, which were central detectors and modulators of inflammation (*n* = 20) [56]. Among older adults with mild cognitive impairment, mindful awareness practice (MAP) exhibited significant reductions in hs-CRP after 9 months for all participants in the MAP group and in IL-6 and IL-β at 3 months for males of MAP, compared to the health education program group (*n* = 55) [57]. The level of TNF-α was not changed by both MBSR [56] and tai chi [58]. Another detrimental neurodegenerative disorder, Parkinson’s disease (PD), also exhibits higher peripheral concentrations of inflammatory markers—including IL-6, TNF, IL-1β, IL-2, IL-10, CRP and regulated upon activation, normal T cell expressed and presumably secreted (RANTES)—according to a meta-analysis that included 25 studies [80]. In an RCT with early-stage PD patients, tai chi practice significantly reduced IL-1β, IL-7, IL-9 compared to usual care; however, the effects were equivalent to brisk walking [59]. For MBI studies with PD patients reporting inflammatory outcomes, there was only one study; therefore, more RCTs are expected to discuss the effects of MBI on inflammation in PD conditions.

Multiple trials sought to determine the potential benefits of MBI for immune modulation in cancer patients. While breast cancer patients undergoing active therapy did not show significant differences in CRP levels by MBSR [60], breast cancer patients after active therapy (e.g., surgery) exhibited significant improvements in immune function by MBIs [61,62,63]. In an RCT involving early-stage breast cancer patients, 8 weeks of MBSR promoted significantly better NK cell activity and lower levels of TNFα, IL-6, and interferon gamma (IFNγ) compared to the control group, who participated in a series of cancer recovery and health education classes (*n* = 124) [61]. In another RCT with breast cancer patients who completed adjuvant chemotherapy and/or radiation therapy, participants were divided into three groups: MBSR, active control (self-instructing MBSR), and no intervention group. Eight weeks of both MBSR training and active control significantly reduced the number of NK cells compared to the no intervention group, while it did not change the levels of IL-6 and IL-8. Within the MBSR group, NK cell activity and CD19^+^ B-lymphocyte number were increased and the percentages of CD3^+^ or CD3^+^8^+^ T lymphocytes were decreased, while IL-6 and IL-8 were not changed at post-intervention compared to baseline (*n* = 166) [62]. Six weeks of MBSR for breast cancer survivors immediately reduced the level of IL-6 after classes in the sixth week; however, there were no differences between groups for changes over time (*n* = 322) [63]. There were two RCTs of MBIs with patients of other cancer types that reported immune-related outcomes. In an RCT involving patients with gastrointestinal cancer, yoga significantly reduced the levels of inflammatory cytokines IL-6 and sTNF-R1 compared to the attention control group. The level of TNF-α was not different between the groups (*n* = 44) [64]. In another RCT on patients with myeloproliferative neoplasm, the level of TNF-α was revealed to be reduced by online yoga training of 12 weeks (*n* = 48) [65]. Most of these MBI studies reported positive or no changes in immune-related outcomes for cancer patients.

Rheumatoid arthritis, an autoimmune disease, has been shown to accelerate immune system aging and reduce immune cell function with chronic low-grade systemic inflammation. Two recent RCTs showed that yoga intervention promoted beneficial effects in reducing inflammation in patients with rheumatoid arthritis. In one RCT, the levels of systemic inflammation markers, including erythrocyte sedimentation rate and CRP, and pro-inflammatory cytokines, such as IL-6, IL-17A, and TNF-α, were lowered, whereas the levels of an anti-inflammatory cytokine transforming growth factor beta (TGF-β) and immunomodulatory marker human leukocyte antigen-G (HLA-G) were increased (*n* = 72) [66]. In another RCT, yoga also significantly reduced the levels of pro-inflammatory cytokines, such as IL-1α, IL-6, and TNF-α (*n* = 166) [67]. Some MBI studies reported immune-related outcomes for not only rheumatoid arthritis patients, but also axial spondyloarthritis patients. Training programs consisted of breathing, exercise, and gradual cold exposure, and meditation significantly reduced the levels of ESR and ASDAS-CRP, but not levels of calprotectin and hs-CRP, after 8 weeks compared to the usual care control (*n* = 24) [68]. In these three RCTs for arthritis patients, MBIs report partially beneficial, at least not harmful, changes in immune-related outcomes among the investigated items.

Studies reported that dysregulated inflammation can be observed in wide range of disease conditions, such as metabolic syndrome [81], myocardial infarction [82], fibromyalgia [83], glaucoma [84], and HIV [85]. In people with metabolic syndrome, yoga-based lifestyle interventions significantly reduced the levels of pro-inflammatory cytokine IL-6 after 2 weeks of intervention, maintaining this reduction until the end of the treatment (12 weeks), although other pro-inflammatory markers, such as TNF-α, remained unchanged (*n* = 260) [26]. In an RCT involving patients with myocardial infarction, qigong practice altered 80 proteins based on proteomic analysis, more than half of which (41 proteins) were associated with immune processes (*n* = 110) [69]. In patients under cardiac rehabilitation, hsCRP levels were reduced in the MBSR group compared to the control group, although the difference was not statistically significant (*n* = 47) [35]. Fibromyalgia patients exhibited a reduction in anti-inflammatory IL-10 levels within a time frame of 8 weeks in usual care, while MBSR intervention successfully maintained IL-10 levels after 8 weeks (*n* = 70) [70]. In patients with glaucoma, 21 days of training of mindfulness meditation significantly reduced the levels of IL-6 and TNF-α, while waitlist control did not (*n* = 82) [71]. In HIV-1 patients, MBSR significantly improved psychological conditions but did not induce statistical improvements in immune-related outcomes (*n* = 177) [72].

The above results indicate that MBIs have positive impacts on immune-modulation in some conditions, suggesting their possible efficacy in treating symptoms of chronic inflammation or dysregulated immune response.

## 5. Underlying Mechanism of MBI: Link between Stress Reduction, Glycemic Control, and Immune Modulation

Studies in both animals and humans have shown that physiological responses to psychological stress are associated with T2DM [86] and immune function [87]. Under stress conditions, the sympathetic nervous system (SNS) and hypothalamus–pituitary–adrenal (HPA) axis are activated. SNS activation increases adrenaline release from the adrenal medulla, inducing energy mobilization and increasing the secretion of cytokines together with other multiple physiological responses. Simultaneously, HPA axis activation induces cortisol secretion from the adrenal cortex into the circulation, which directly reduces insulin secretion [88], increases glucose levels in the systematic circulation, and suppresses inflammatory responses [89,90]. Chronic exposure to cortisol increases susceptibility to hyperglycemia and diabetes mellitus [91]. Moreover, patients with chronically elevated glucose levels, such as those with T2DM, exhibit low-grade inflammation through multiple pathways [92,93]. Indeed, a large epidemiological study involving 3500 individuals revealed that people with T2DM had a flatter slope of cortisol level during the day and greater evening levels of cortisol compared with healthy people [94].

Psychological stress-induced secretions of hormones and neurotransmitters, such as cortisol, epinephrine, and norepinephrine, affect immune cells, causing changes in the immune system during stress [95,96]. Chronic exposure to stress can promote negative systemic changes in the immune system [97]. For instance, psychological stress, such as negative social interactions, have been linked to increased inflammation [98]. Moreover, aging coupled with chronic stress exacerbates immune senescence [99]. Under chronic stress, the negative feedback loop for cortisol is disabled, resulting in a dysregulated immune system, i.e., increased inflammation and compromised immune response [99]. For instance, caregivers providing long-term care exhibit lower antibody and cell-mediated immune responses after vaccination [100,101].

Considering the effects of psychological stress on blood glucose levels and immune function as described above, stress management through MBI may play a protective role against the stress-induced acceleration of diabetes, systemic inflammation, or immune system dysregulation. Despite the varying types of MBI, the key benefits of MBI commonly include stress reduction, mood improvements, and improved sleep [102,103,104,105]. These beneficial changes associated with stress reduction may be associated with reduced activity of the SNS together with the HPA axis, which results in an improvement in immune modulation and lower inflammation [87,106], as well as decreases in blood glucose levels [11,12]. Recent studies showed the potential diabetogenic effect of COVID-19 and the increase in the risk of severe COVID-19 caused by diabetes. The beneficial modulating effects of MBI on blood glucose and the immune system suggest that MBI can be used as a complementary approach in combination with regular therapies to manage COVID-19 or long COVID symptoms.

## 6. Utilization of MBI for COVID-19 and Long COVID

### 6.1. Symptoms of COVID-19 and Long COVID

According to a recent systematic review and meta-analysis of 51 studies with 18,917 patients, survivors of COVID-19 exhibit neuropsychiatric symptoms, including sleep disturbances (27.4%), fatigue (24.4%), objective cognitive impairment (20.2%), anxiety (19.1%), and post-traumatic stress (15.7%) at a mean of 77 days (range 14–182 days) after COVID-19 [107]. Aside from mental symptoms, various persistent physical symptoms, such as shortness of breath, chest pain, altered smell and taste, cough, myalgia, diarrhea, and organ dysfunction, were observed following COVID-19 infection [108,109]. MBI often includes meditation, breathing training, and focused body movements, which could potentially relieve multiple symptoms of COVID-19 or long COVID, such as shortness of breath, fatigue, anxiety, sleep disturbance, cognitive impairment, and post-traumatic stress [104,110,111,112,113,114,115].

### 6.2. Link between Diabetes, Immunity, and Mental Health in the COVID-19 and Long COVID

#### 6.2.1. Mental Health and Diabetes in the COVID-19 and Long COVID

Among the general population, COVID-19 lockdowns have led to multiple psychological symptoms, such as anxiety, depression, and anger [116]. People with diabetes experience symptoms of psychological distress, such as hopelessness, anger, or frustration, which can affect patients’ health behaviors, for instance, causing a lack of self-care or reduced compliance with treatment [117]. These negative emotions, which are called diabetes distress, arise from living with diabetes and the burden of self-management. During COVID-19 or post-COVID, lockdown-mediated social isolation, uncertainty, anxiety and reduced physical activity can aggravate diabetes-specific emotional distress [118], which may decrease compliance to therapeutic regimens and decreased glycemic control [119]. Poor mental health and socioeconomic issues caused by COVID-19 or post-COVID may increase the risk of T2DM, given the previously known T2DM risk factors, such as stressful working conditions, traumatic events, depression, conflict with others, and low socioeconomic status [120].

#### 6.2.2. Mental Health and Immunity in the COVID-19 and Long COVID

Poor mental health states, such as depression, anxiety, and stress, have been associated with poor immune functions such as low-grade inflammation. In a meta-analysis that compared 5166 patients with depression and 5083 controls, patients with depression exhibited a significantly higher pro-inflammatory state (i.e., higher levels in CRP, L-3, IL-6, IL-12, IL-18, sIL-2R, and TNFα) than controls [121]. A meta-analysis investigating the relationship between anxiety and immune features based on 41 studies found significant differences in pro-inflammatory cytokines, including interleukin-1ꞵ, IL-6, and tumor necrosis factor-α, between healthy controls and people with anxiety disorders [122]. Another meta-analysis, examining the effects of acute stress on salivary inflammation from 33 studies with 1558 participants, showed significantly increased salivary inflammatory markers, such as IL-6, IL-10, TNF-alpha, and IFN-gamma, in response to stress [123]. COVID-19 can affect mental health not only through infection itself but also socioeconomically, which may increase susceptibility to infection and difficulty in recovering from a diseased state.

#### 6.2.3. Improvements in Mental Health through MBI

It is well-established that MBI techniques (e.g., meditation, yoga, qigong, etc.) induce the relaxation of the body and mind by activating the parasympathetic nervous system (PNS), leading to beneficial effects on mental health for a variety of different people (the elderly, students, patients with post-traumatic stress disorder (PTSD) or depression, etc.). A meta-analysis involving 1076 participants from 19 studies found that mindfulness meditation interventions improved depressive symptoms in older adults [124]. Meditation techniques are also valid for other mental health issues. In fact, a meta-analytic review that compared the effects of meditation and relaxation therapies on anxiety, based on 14 RCTs with 862 participants suffering from anxiety disorders or high-trait anxiety, revealed that meditation promoted slightly better outcomes for anxiety symptoms than relaxation, which might remain more effective at 12-month follow-up [112]. In addition, meditation and yoga seem to be promising complementary approaches for PTSD patients according to a meta-analysis of 19 RCTs with 1173 patients [115]. Considering the link between mental health, diabetes and the immune system, it is expected that better mental health by MBI might contribute to reducing the occurrence of diabetes, its complications, decreasing infection rates of infectious diseases, as well as increasing recovery rates from infections.

### 6.3. Effects of MBI on General Health in Those with COVID-19 and Long COVID

#### 6.3.1. Improvements in Dyspnea, Anxiety, Depression, and Quality of Life following MBI for COVID-19 Patients/Survivors

Since the COVID-19 outbreak, studies on MBI in patients with COVID-19, other diseases, and the general public have contributed to investigating the health benefits of MBI, which can be applied through self-health management techniques. In general, several MBIs often include some common components, for example, breathing practices. This common feature of MBI makes it an attractive approach for complementary therapy against COVID-19 and long COVID, as its major symptoms have been associated with pulmonary function and breathing. COVID-19 can cause severe pulmonary disease. One study showed that among the 156 participants across five large Norwegian hospitals, one-third of participants experienced a reduction in oxygen uptake (V’O2peak < 80%) during cardiopulmonary exercise testing 3 months after COVID-19 discharge [125]. Therefore, a proper breathing practice may contribute to a reduction in or elimination of dyspnea. Moreover, given that deep breathing usually activates the PNS, it may also reduce anxiety and improve one’s quality of life. The followings are results of RCTs investigating the effects of breathing on the health condition of COVID-19 patients or survivors.

An RCT conducted in the COVID-19 clinic of a tertiary hospital with 44 patients found that deep breathing exercises in the triflo group exhibited a significantly shorter hospitalization time, lower anxiety levels, higher oxygen saturation levels (SpO2), and a higher quality of life compared to the usual care group [126]. Among post-discharge COVID-19 patients, moderate to high-intensity aerobic (e.g., upper or lower limb ergometry, elliptical or treadmill, 20–60 min/session) and breathing exercises (10 min/session) for 5 weeks (3 sessions/week) significantly improved cardiorespiratory fitness [127]. In an RCT involving 120 COVID-19 survivors, a 6-week telerehabilitation program comprising breathing control, thoracic expansion, aerobic exercise, and lower limb muscle strength (LMS) promoted a significantly better 6 min walking distance, LMS, and physical health-related quality of life compared to controls [128]. In another study comprising 144 patients diagnosed with COVID-19, an intervention with psychological support and breathing exercise significantly exhibited less depression and anxiety and greater perceived social support level compared to the control group, after a 10-day intervention [129]. In another study, COVID-19 patients in home confinement over the last 40 days were randomized into breathing exercise or control groups. They were asked to perform breathing exercises for 10–30 min daily over 7 days depending on the Borg evaluation scale, which reflects the intensity of exercise or workload. Accordingly, the breathing group but not the control group showed a significant decrease in Borg scale scores and dyspnea and a significant increase in exercise capacity (measured using the duration of 6 min walking test (6MWT)) and peripheral muscle performance of the lower limbs (measured using the 30 s sit-to-stand test). Moreover, there were significant between-group differences in all of the above-mentioned measures [130]. In an RCT involving 72 elderly patients with COVID-19, 6-week respiratory rehabilitation training with respiratory muscle training, cough exercises, diaphragmatic training, stretching exercises, and home exercises significantly improved pulmonary function (measured using forced expiratory volume at 1 s (FEV1(L)), forced vital capacity (FVC (L)), FEV1/FVC%, diffusing lung capacity for carbon monoxide (DLCO%), exercise capacity (measured using the 6MWT), and quality of life [131]. Internet-based online MBI also exhibited a beneficial effect on the mental health of patients. In a 2-week RCT investigating 26 COVID-19 patients, the daily practice of the internet-based integrated intervention, which included breath relaxation training and body scan (mindfulness) significantly reduced anxiety and depression [132].

#### 6.3.2. Effects of MBI for Patients with Other Diseases, Health Care Professionals, and the Elderly during the COVID-19 Pandemic

During the COVID-19 pandemic, several cities worldwide experienced a health care crisis, which impacted the treatment of patients with other disease besides COVID-19 and public health in general. Given that MBI is non-pharmacological and easy to access, it can be used as a self-health management technique not only for COVID-19 patients/survivors but also a wide range of populations, including healthy individuals. In fact, an RCT involving 101 obstetrics and gynecology patients during the COVID-19 pandemic showed that a mobile meditation app significantly reduced perceived stress, depression, anxiety, and sleep disturbance compared to standard care [133]. During the pandemic, health care professionals experienced unprecedented work pressure, causing mental health issues for such populations. An RCT investigating 155 physicians and advanced practice providers who were lonely (50%) and had sleep problems (97%) found that 4 weeks of daily meditation training significantly reduced loneliness and improved sleep quality [134]. Another RCT that investigated community-dwelling older adults revealed that 3.5 weeks of cognitive–behavioral and mindfulness practice significantly reduced levels of depression and loneliness compared to the waitlist control group during the pandemic [135]. Moreover, another RCT exploring the health benefits of tai chi for older adults during the pandemic showed that a 10-week tai chi intervention significantly decreased perceived stress compared to the control group (*n* = 30) [136].

### 6.4. Practical Implications of MBI for COVID-19 and Long COVID

As MBIs are non-invasive/non-pharmacological interventions, and their intensity, frequency and education details can be customized according to the target populations, they are currently widely used with target-modified versions for various target populations, including patients with diabetes, hypertension, cancer, affective disorders, PTSD, etc. A wide age range is also applicable as the methods can be modified for the target age. There are no gender limitations in the application for these methods. Once a method is properly learned from a certified expert of a certain MBI program, one can freely practice by oneself for one’s own health management for a lifetime. The personal and social burden in health management and recovery increased throughout the pandemic. Organizations for public health can communicate the benefits, safety, precautions of MBIs more actively and support MBI education for the public in order to improve individuals’ mental and physical health condition at home, especially in times of difficulty in use for public health services during the pandemic.

## 7. Limitations of the Study

Multiple limitations exist in the current study. As this was not a systematic review or meta-analysis, statistical conclusions could not be made. Investigations of both glycemic and immune-related outcomes within a single study have rarely been performed; therefore, the papers included in the current study mostly report outcomes related to only one aspect (either glycemic or immune-related outcomes). In the included papers, there is a wide variety of MBI methods and participant characteristics. Therefore, a specific MBI that has beneficial effects on one health aspect (e.g., glycemic control) does not guarantee its effects for another aspect (e.g., immunity) as well. For future studies, RCTs handling both aspects within specified populations should be performed.

## 8. Conclusions

Although much of the relationship between COVID-19 and diabetes still remains unclear, evidence regarding such an association has emerged. COVID-19 may induce diabetes, while pre-existing diabetes can exacerbate COVID-19 symptoms. MBI reduces psychological stress and can improve mental and physical health. Accumulated studies suggest that MBI may have positive effects on glycemic control and immune modulation potentially through stress reduction, followed by reduced HPA axis/SNS activation and increased PNS activity. This suggests that MBI may potentially improve the prognosis of COVID-19, which may cause diabetes or exacerbate symptoms among those with pre-existing diabetes.

## Figures and Tables

**Table 1:** The effects of Mind–Body Intervention on blood-glucose-related biochemical measures. Randomized controlled trials over the last 5 years regarding the effects of Mind–Body Intervention on blood-glucose-related biochemical measures are summarized.

Refs.	Study Type	Country	Participants	Number of Participants	Gender	Age	Intervention	Control	Duration	Intervention Frequency	Measurements at	Outcomes (Blood-Glucose-Related Biochemical Measures)
[22]	RCT	USA	Patients with T2DM	48 (24 for each group)	63% Female	56	Iyengar yoga	Standard Exercise (walking program)	3 months	60 min/session, 2 sessions/week.	Pre, post (3 months), follow-up (6, 9 months)	Yoga: ↓ FBG (6 vs. 9 months), ↔ HbA1c (*p* = 0.06, 3 vs. 6 months). Walking: ↔ FBG, HbA1c.
[23]	RCT	China	Patients with T2DM	87 (34 for qigong, 24 for tai chi, 29 for control)	46% Female	60	Group1: Fitness qigong, Group2: Tai chi	Group3: Stretching	3 months	1 h guided session/week. daily home practice	Pre, post (3 months)	Qigong (vs. control): ↔ FPG, ↔ HbA1c. Tai chi (vs. control): ↔ FPG, ↑ HbA1c.
[24]	RCT	USA	Patients with T1DM	48 (16 for each group)	50% Female	16–20	Group1: MBSR, Group2: CBSM	Group3: Diabetic support	9 weeks	90–120 min/session, 1 session/week	Pre, post (3 months), Follow-up (6 months)	HbA1c (pre vs. post): ↔ (MBSR), ↔ (CBSM), ↓ (diabetic support).
[25]	RCT	China	Adults with hypertension and two modifiable cardiovascular disease risk factors	246 (82 for each group)	55% Female	64	Group1: Tai chi, Group2: brisk walking	Group3: Usual activity	9 months	For initial 3 months: 30 min/day, at least 5 days/week. For the next 6 months: daily home-based practice	Pre, post (3 months), follow-up (6, 9 months)	Tai chi (vs. control, at 9 months): ↓ FBG, HbA1c. Brisk walking (vs. control, at 9 months): ↓ HbA1c. Tai chi (vs. brisk walking at 6, 9 months): ↓ FBG, HbA1c.
[26]	RCT	India	Adults with metabolic syndrome	260 (130 for each group)	68% Female	38	YBLI	DI	12 weeks	For the first 2 weeks: 2 h guided session/day, 5 days/week. For the next 10 weeks: same interventions at home.	Pre, during intervention (2 weeks), post (12 weeks)	↓ FPG (baseline vs. 2 (12) weeks, in both groups, no group difference)
[27]	RCT	China	Adults with metabolic syndrome	54 (27 for each group)	48% Female	64	Tai chi	Usual daily activity	12 weeks	1 h/session, 2 sessions/week. 30 min home practice, 3 times/week	Pre, post (12 weeks)	↔ FBG, HbA1c (between group)
[28]	RCT	India	Pre-diabetic women	37 (22 for diabetic yoga protocol, 15 for control)	100% Female	53	Diabetic yoga protocol	Waitlist	3 months	32 min daily session	Pre, post (3 months)	↓ HbA1c, FPG
[29]	RCT	USA	Women with BMI ≥ 25	86 (42 for MBSR, 44 for HE)	100% Female	45	MBSR	HE	8 weeks	2.5 h instructor-led weekly session for 8 weeks (MBSR, HE). One 6 h retreat (MBSR). 25–30 min daily home practices (MBSR)	Pre, post (8 weeks), follow-up (16 weeks)	↓ FPG (8, 16 weeks)
[30]	RCT	USA	Adolescent girls with overweight/obesity, family history of diabetes, and elevated depressive symptoms	33 (17 for mindfulness group, 16 for cognitive–behavioral program)	100% Female	15	Mindfulness-based group intervention	Cognitive–behavioral intervention	6 weeks	1 h session/week. 10 min daily homework	Pre, post (6 weeks), follow-up (6 months)	Mindfulness (vs. Cognitive behavioral intervention) Fasting insulin: ↓ (post), ↔ (follow-up). FBG: ↔ (post, follow-up). Insulin resistance: ↓ (post), ↔ (follow-up).
[31]	RCT	China	Older adults with high risk of ischemic stroke	170 (85 for each group)	59% Female	61	Tai chi	Usual physical activity	12 weeks	60 min/session, 5 sessions/week	Pre, post (12 weeks), follow-up (24 weeks)	Tai Chi (vs. Usual physical activity): ↓ FBG.
[32]	RCT	India	Non-diabetic offspring of T2DM parents	57 (28 for yoga, 29 for control)	n.d.	26	Yoga	Control	8 weeks	1 h/session, 5 sessions/week	Pre, post (8 weeks)	Within group: Yoga: ↓ FPG, OGTT post 2 h glucose, fasting insulin, insulin resistance. Control: no changes. (between group, ANCOVA). Significant changes in FPG, OGTT post 2 h glucose, fasting insulin, insulin resistance.
[33]	RCT	Taiwan	Long-term care residents	120 (60 for each group)	65% Female	79	Mindfulness program (meditations + education + exercise)	Usual care	9 weeks	1.5 h session/week	Pre, post (3 months)	Mindfulness: ↓ HbA1c. Control: no changes.
[34]	RCT	UK	Patients undergoing cardiac rehabilitation following acute coronary events	60 (25 for yoga with usual care, 35 for usual care alone)	32% Female	35–80	Yoga with usual care	Usual care	3 months	75 min/session, 2 group sessions/week	Pre, post (3 months)	↔ FBG (between group)
[35]	RCT	USA	Cardiac patients	47 (31 for MBSR, 16 for control)	38% Female	59	MBSR	Usual care	8 weeks	2.5 h session/week. One 6.5 h retreat.	Pre, post (3 months), follow-up (9 months)	↔ HbA1c

Values in age column represent mean or range of age. Arrows indicate the following: ↓, decrease; ↑, increase; ↔, no change. Abbreviations: RCT, randomized controlled trial; T2DM, type 2 diabetes mellitus; FBG, fasting blood glucose; HbA1c, glycated hemoglobin; FPG, fasting plasma glucose; T1DM, type 1 diabetes mellitus; MBSR, mindfulness-based stress reduction; CBSM, cognitive–behavioral stress management; YBLI, yoga-based lifestyle; DI, dietary intervention; HE, health education; n.d., no data; OGTT, oral glucose tolerance test.

**Table 2:** Effects of the Mind–Body Intervention on immune function. Randomized controlled trials over the recent 5 years regarding the effects of the Mind–Body Intervention on immune function-related measures were summarized.

Refs.	Study Type	Country	Participants	Number of Participants	Gender	Age	Intervention	Control	Duration	Intervention Frequency	Measurements at	Outcomes (Immune Function-Related Measures)
[46]	RCT	USA	Healthy adults	413 (126 for MBSR, 124 for aerobic exercise, 130 for control)	76% Female	50	Group1: MBSR, Group2: Aerobic exercise	Group3: Waitlist	8 weeks	2.5 h sessions/week, One half-day retreat	Pre, post (8 weeks), Follow-up (17 weeks)	MBSR: ↓ CRP (17 weeks), ↔ IL-6, IP-10. Aerobic exercise: ↓ IP-10 (8, 17 weeks).
[47]	RCT	USA	Adults with moderate to high levels of stress	Study1: 153 (58 for MA, 58 for MO, 37 for control). Study2: 137 (54 for MA, 53 for MO, 30 for control).	67% Female	Study1: 32. Study2: 38.	Group1: MA, Group2: MO	Group3: Stress management	Study1: 2 weeks. Study2: 8 weeks.	Study1: Smartphone application, 20 min audio training + 3–10 min home practice/day. Study2: 2.5–3 h in-person session/week, 1 day-long retreat, 45 min of daily home practice.	Pre, post	Study1, 2: No group differences in CRP level.
[48]	RCT	Netherlands	Healthy adults	49 (23 for MBSR, 26 for control)	84% Female	22	MBSR	Waitlist	8 weeks	2.5 h session/week. Daily home practice (30–60 min).	Pre, post (8 weeks)	↔ IL-1β, IL-6, TNF-α; ↓ IL-8
[49]	RCT	USA	Adolescents	38 (21 for MBSR-T, 17 for control)	42% Female	14	MBSR-T	Usual care	4 weeks	10–20 min/session, 2 sessions/week. On-your-own practices.	Pre, post (4 weeks)	↔ CRP, IL-6
[50]	RCT	Hongkong	Cognitively healthy older people	48 (22 for qigong, 26 for stretching)	69% Female	64	Qigong	Stretching	12 weeks	2 h/session, total 18 sessions, 30 min-daily home practices	Pre, post (12 weeks)	↓ IL-6
[51]	RCT	Germany	Ostensibly healthy adults	332 (80 for training cohort (TC)1, 81 for TC2, 81 for TC3, 90 for control)	58% Female	20–55	Contemplative mental training (Presence, affect, perspective modules)	No training	3 × 3 month	3-day retreat, 2 h group session/week, 30 min daily online home practice	Pre, during intervention (3-, 6-month), post (9-month)	↔ IL-6, hs-CRP (no group-level effect of training); ↓IL-6, hs-CRP (presence module, male, high inflammatory load)
[52]	RCT	Spain	Healthy subjects	43 (25 for qigong, 18 for control)	79% Female	18–21	Taoist qigong practice	Control	1 month	3 days of group practice (25–30 min × 2)/week, total 15~20 sessions, Home practices.	Pre, post (1 month)	↑ B lymphocyte (number, %); ↓ NK cells (%)
[53]	RCT	UK	Students with exam stress	54 (27 for each group)	70% Female	17–21 (44%); 22–30 (44%); 31+ (11%)	Mindfulness course, + mental health support	Mental health support	8 weeks	75–90 min session/week	Pre, post (8 weeks)	↔ Cortisol, CRP, IL-8, TNF-α, %CD4+ T lymphocytes, %CD8+ T lymphocytes, %CD19+ B lymphocytes, %CD14+ monocytes, %NK cells
[54]	RCT	USA	Patients with major depression	87 (48 for yoga, 39 for health education)	84% Female	45	Yoga	Health education	10 weeks	80 min/session, 2 sessions/week	Pre, during intervention (3 weeks), Post (10 weeks)	↓ IL-6; ↔ TNF-α, CRP
[55]	RCT	USA	Patients with generalized anxiety disorder	70 (42 for MBSR, 28 for SME)	46% Female	39	MBSR	SME	8 weeks	One session/week. A single weekend retreat. Daily home practice.	Pre, post (8 weeks)	Intervention: ↓ IL-6, TNF-α Control: ↑ IL-6, TNF-α
[56]	RCT	Czech	Older adults with MCI	20 (12 for MBSR, 8 for control)	65% Female	74	MBSR	Cognitive training	8 weeks	2.5 h group session/week. 30–50 min home practices/day. One 6 hr retreat	Pre, post (8 weeks)	↓ activation of monocytes (CD86 in CD14^+^ and CD14^+^CD16^+^ monocytes); ↔ proportion of monocyte subsets, phagocytic activity of the PBMCs, TNF-α, IL-6, CRP
[57]	RCT	Singapore	Older adults with MCI	55 (28 for mindful practice, 27 for control)	75% Female	71	Mindful awareness practice	Health education program	9 months	For first 3 months, 1 h session/week. For the next 6 months, 1 session/month.	Pre, during intervention (3-month), post (9-month)	↓ hs-CRP (total, 9 months), IL-6 (males, 3 months), IL-1β (males, 3 months).
[58]	RCT	Thailand	Older adults with amnestic MCI	66 (33 for tai chi, 33 for control)	86% Female	68	Tai chi	Usual care	6 months 3 weeks	3 group sessions/week for the initial 3 weeks. 3 home sessions/week for the following 6 months.	Pre, post (6 months 3 weeks)	↔ TNF-α, IL-10
[59]	RCT	China	Early-stage PD patients	95 (32 for tai chi, 31 for brisk walking, 32 for usual care)	39% Female	62	Tai chi	Brisk walking or usual care	1 year	60 min/session. 2 sessions/week.	Pre, during intervention (6 months), Post (12 months)	(T ai chi vs. usual care) ↓ IL-1β, IL-7, IL-9; ↔ IL-1RA, IL-2, IL-4, IL-6, IL-10, IL-12, IL-15, IL-17A, IL-8, IFN-γ, TNF-α;↑ IL-13. (T ai chi vs. brisk walking) ↔ IL-1β, IL-1RA, IL-2, IL-4, IL-6, IL-7, IL-9, IL-10, IL-12, IL-15, IL-17A, IL-8, IFN-γ, TNF-α; ↑ IL-13
[60]	RCT	Iran	Breast cancer patients undergoing chemotherapy or surgery	51 (27 for MBSR, 24 for control)	100% Female	45	MBSR	Usual care	8 weeks	90 min session/week.	Pre, post (8 weeks)	No significant differences between groups in CRP levels after the intervention.
[61]	RCT	USA	Women with early-stage breast cancer after the surgery	124 (63 for MBSR, 61 for ACC)	100% Female	28–75	MBSR	ACC	8 weeks	MBSR: 2.5 h/week, one additional 6-hr retreat after the fifth week, ACC: 2.5 h/week	Pre, during the intervention (4 weeks), post (8 weeks), follow-up (1- and 6-month post intervention)	↑ Restoration of NKCA, IFNγ; ↓ TNFα, IL-6
[62]	RCT	Sweden	Breast cancer survivors	166 (62 for MBSR, 52 for active controls, 52 for non-MBSR)	100% Female	n.d.	Group1: MBSR, Group2: active controls (self-instructing MBSR)	Group3: non-MBSR (no intervention)	8 weeks	MBSR: 2 h group guided session/week, 20 min daily home sessions for other days. Active control: 20 min daily home sessions	Pre, post (8 weeks)	MBSR (vs. No intervention): ↓ NK cell number; ↔ IL-6, IL-8 Active control (vs. No intervention): ↓ NK cell number, % NK cell; ↔ IL-6, IL-8 Pre vs. post (within MBSR): ↑ NK-cell activity, CD19+B-lymphocyte number, %CD19+B-lymphocyte; ↓ % CD3+T-lymphocyte, % CD3+8+T-lymphocyte; ↔ IL-6, IL-8
[63]	RCT	USA	Breast cancer survivors	322 (167 for MBSR, 155 for control)	100% Female	57	MBSR (BC)	Usual care	6 weeks	2 h guided in-person session/week. 15–45 min daily practice.	Pre and post session at 1 and 6th week	Immediate short-term effects following the MBSR class: ↓ IL-6 (at 6 weeks) No significant differences between groups for change over time in IL-6
[64]	RCT	USA	Patients with gastrointestinal cancer	44 (23 for yoga skills training, 21 for attention control)	52% Female	58	Yoga skills training	Attention control (empathic attention with home diaries)	14 weeks	30 min/session, 4 guided sessions during chemotherapy at weeks 2, 4, 6, 8. Daily home practices with 16 min audio recording of the training	Pre, during intervention (10 weeks)	↓ IL-6, sTNF-R1; ↔ TNF-α
[65]	RCT	USA	Patients with myeloproliferative neoplasm	48 (27 for online yoga, 21 for control)	94% Female	57	Online yoga	Normal activity	12 weeks	60 min/week	Pre, post (12 weeks)	↓ TNF-α
[66]	RCT	India	Patients with rheumatoid arthritis	72 (36 for each group)	78% Female	44	Yoga with conventional therapy	Conventional therapy only	8 weeks	120 min/session, 5 sessions/week	Pre, post (8 weeks)	↓ ESR, CRP, IL-6, IL-17A, TNF-α; ↑ TGF-β, HLA-G
[67]	RCT	India	Patients with rheumatoid arthritis	166 (83 for each group)	79% Female	42	Yoga	Control	12 weeks	30 min/session, 3 sessions/week	Pre, post (12 weeks)	(yoga vs. control, at 12 weeks) ↓ IL-1α; ↔ IL-6, NF-α. (baseline vs. 12 weeks, within yoga) ↓ IL-1α, IL-6, TNF-α
[68]	RCT	Netherlands	Patients with moderately active axial spondyloarthritis	24 (13 for an intervention, 11 for control)	37.5% Female	35	Training program (breathing exercises, gradual cold exposure, meditation)	Usual care	8 weeks	2 group sessions/week for the first 4 weeks. 1 group session/week for the second 4 weeks. Daily home practice.	Pre, during intervention (4 weeks), post (8 weeks)	Intervention: ↓ ESR, ASDAS-CRP; ↔ calprotectin, hs-CRP Control: ↔ ESR, ASDAS-CRP, calprotectin, hs-CRP
[26]	RCT	India	Patients with metabolic syndrome	260 (130 for each group)	68% Female	38	Yoga-based lifestyle intervention	Dietary intervention	12 weeks	For first 2 weeks, 2 hr/day under direct supervision. For the next 10 weeks, home intervention.	Pre, during intervention (2 weeks), post (12 weeks)	[baseline vs. 2 (or 12) weeks, within yoga] ↓ IL-6; ↔ TNF-α
[69]	RCT	China	Patients with myocardial infarction	110 (56 for qigong, 54 for physical exercise)	34% Female	60	Qigong-based cardiac rehabilitation program	Aerobic exercise	12 weeks	45 min/session, 2 sessions/week	Pre, post (12 weeks)	Proteomic analysis: qigong-induced variations in the expression of 80 proteins linked to regulation of the metabolic process (38 proteins), immune process (41 proteins), and extracellular matrix reorganization (13 proteins)
[35]	RCT	USA	Patients under cardiac rehabilitation	47 (31 for MBSR, 16 for control)	38% Female	59	MBSR	Usual care	8 weeks	2.5 h session/week. one 6.5 h retreat.	Pre, post (3 months), follow-up (9 months)	↔ hsCRP (3, 9 months)
[70]	RCT	Spain	Patients with fibromyalgia	70 (35 for each group)	100% Female	53	MBSR	TAU	8 weeks	2 h group session/week. 45 min home practices/day. one 6 hr retreat	Pre, post (12 months)	MBSR: ↔ IL-10. TAU: ↓ IL-10
[71]	RCT	India	Patients with primary open angle glaucoma	82 (40 for meditation, 42 for control)	44% Female	57	Mindfulness meditation	Waitlist	21 days	Daily	Pre, post (21 days)	Intervention: ↓ IL-6, TNF-α. Control: ↔ IL-6, TNF-α.
[72]	RCT	USA	Patients with HIV-1 infection	177 (89 for MBSR, 88 for control)	3% Female	40	MBSR	Health education	8 weeks	(MBSR) 2.5 h session/week. 8 h silent retreat at the sixth week. Home practice. (control) 1.5 h session/week.	Pre, post (3 months), follow-up (12 months)	No within group differences in CD4 T cell number, HIV-1 viral load, IL-6, and hsCRP at 3, 12 months.

Values in age column represent mean or range of age. Abbreviations: RCT, randomized controlled trial; MBSR, mindfulness-based stress reduction; CRP, C-reactive protein; IL, interleukin; IP-10, interferon-gamma-inducible protein-10; MA, mindfulness training with attention monitoring and acceptance skills; MO, mindfulness training with monitoring only; MBSR-T, MBSR for Teens; hs-CRP, high-sensitivity CRP; NK, natural killer; TNF-α, tumor necrosis factor-alpha; SME, stress management education; MCI, mild cognitive impairment; PBMC, peripheral blood mononuclear cell; PD, Parkinson’s disease; ACC, a series of cancer recovery and health education classes; NKCA, natural killer cell activity; IFN-γ, interferon-gamma; sTNF-R1, soluble tumor necrosis factor receptor 1; ESR, erythrocyte sedimentation rate; HLA-G, human leukocyte antigen-G; ASDAS, ankylosing spondylitis disease activity score; HIV, human immunodeficiency virus. Arrows indicate the following: ↓, decrease; ↑, increase; ↔, no change.

## Supplementary Materials

The following supporting information can be downloaded at: https://www.mdpi.com/article/10.3390/ijerph19116618/s1, Figure S1: A PRISMA Flow diagram to describe study selection process; Table S1: Quality assessment of randomized controlled trial by the NIHLBI guidelines with minor modifications for selected studies.
