# Supplementary material for: Utilization of Mind–Body Intervention for Integrative Health Care of COVID-19 Patients and Survivors
Source: Int J Environ Res Public Health. 2022 May 29;19(11):6618. doi: 10.3390/ijerph19116618 (PMC9180827; doi:10.3390/ijerph19116618)
Supplement: Supplementary file 1 [file ijerph-19-06618-s001.zip › ijerph-1695988-supplementary.pdf]

Supplementary Figure S1. A PRISMA Flow diagram to describe study selection process.

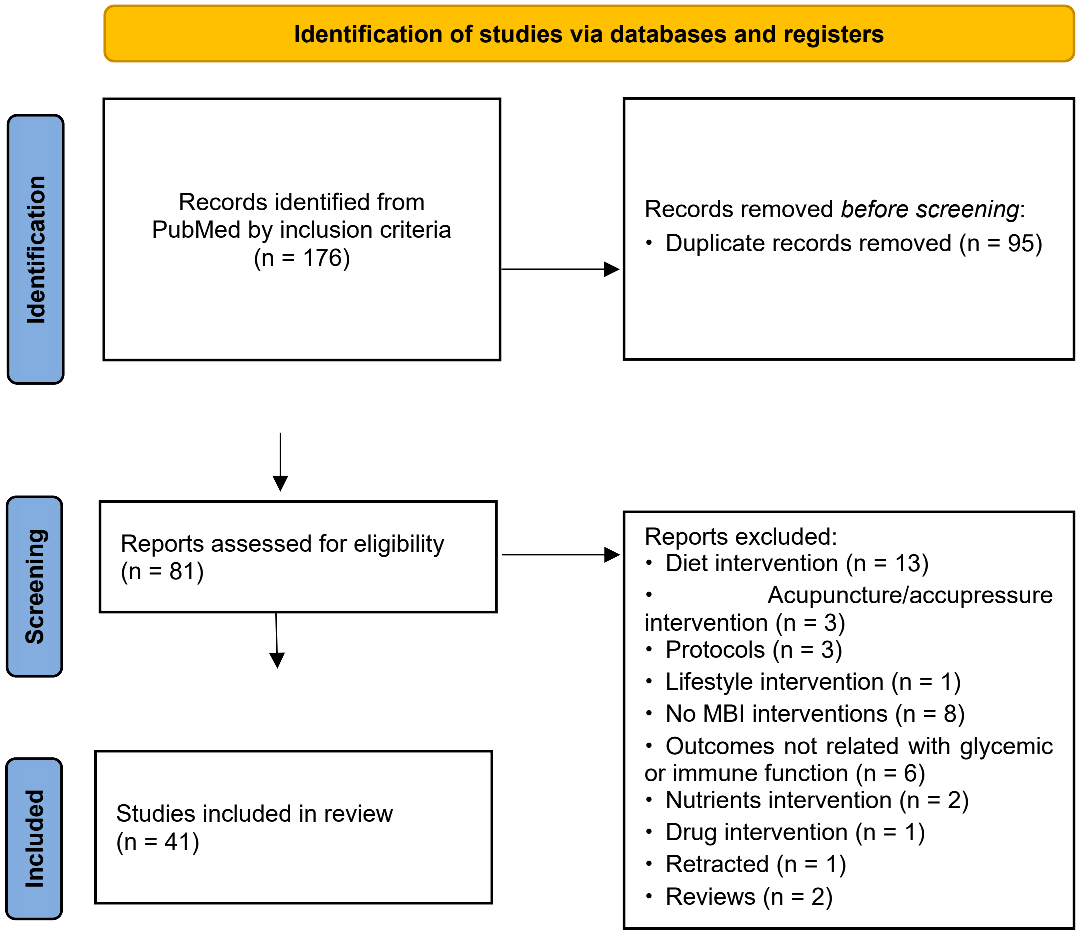

Supplementary Table S1. Quality assessment of randomized controlled trial by the NIHLBI guidelines with minor modifications for selected studies.

Study	Item 1	Item 2	Item 3	Item 4	Item 5	Item 6	Item 7	Item 8	Item 9	Item 10	Item 11	Total
[1]	1	1	1	1	0	1	1	1	0	1	0	8
[2]	1	1	1	1	0	1	1	1	0	1	0	8
[3]	1	1	1	1	1	1	1	1	0	1	1	10
[4]	1	1	1	0	1	1	1	1	1	1	1	10
[5]	1	1	1	0	1	1	1	1	1	1	0	9
[6]	1	1	1	0	1	1	1	1	0	1	0	8
[7]	1	1	1	0	0	1	0	1	0	1	0	6
[8]	1	1	1	0	0	1	0	1	1	1	1	8
[9]	1	1	1	1	1	1	1	1	0	1	1	10
[10]	1	1	1	0	1	1	0	1	1	1	0	8
[11]	1	1	1	0	1	0	1	1	0	1	1	8
[12]	1	1	1	1	1	1	0	1	0	1	0	8
[13]	1	1	1	1	1	0	0	1	1	1	0	8
[14]	1	1	1	1	1	1	1	1	0	1	1	10
[15]	1	1	1	1	1	1	1	1	0	1	0	9
[16]	1	1	1	1	1	1	1	1	0	1	0	9
[17]	1	1	1	0	1	1	0	1	0	1	0	7
[18]	1	1	1	1	1	0	0	1	0	1	0	7
[19]	1	1	1	1	1	1	0	1	0	1	1	9
[20]	1	1	1	0	1	1	1	1	0	1	1	9

[21]	1	1	1	1	1	0	1	1	1	1	0	9
[22]	1	1	1	1	1	1	1	1	0	1	0	9
[23]	1	1	1	0	1	1	0	1	0	1	0	7
[24]	1	1	1	1	1	0	0	1	1	1	1	9
[25]	1	1	1	1	1	0	0	1	1	1	0	8
[26]	1	1	1	1	1	1	1	1	1	1	0	10
[27]	1	1	1	1	1	1	1	1	0	1	0	9
[28]	1	1	1	1	0	1	0	1	1	1	1	9
[29]	1	1	1	1	1	1	1	1	0	1	1	10
[30]	1	1	1	1	1	1	0	1	0	1	1	9
[31]	1	1	1	1	1	1	0	1	0	1	0	8
[32]	1	1	1	1	1	1	0	1	1	1	1	10
[33]	1	1	1	1	1	0	0	1	1	1	1	9
[34]	1	1	1	1	1	1	0	1	1	1	0	9
[35]	1	1	1	1	0	1	1	1	0	1	1	9
[36]	1	1	1	1	1	1	0	1	0	1	0	8
[37]	1	1	1	0	0	0	1	1	0	1	1	7
[38]	1	1	1	1	1	0	0	1	0	1	0	7
[39]	1	1	1	1	1	0	0	1	1	1	0	8
[40]	1	1	1	1	1	1	0	1	1	1	1	10
[41]	1	1	1	1	1	1	1	1	1	1	0	10

Details of each item for quality assessment: 1. RCT; 2. Adequate randomization method; 3. Similarity of groups at baseline; 4. Drop-out rate less than 20% at end point; 5. Differential drop-out rate less than 15%; 6. Adherence to intervention protocols; 7. Similar background intervention; 8. Valid and reliable

outcome measurement; 9. Power calculation; 10. Pre-specified outcomes; 11. Intention-to-treat analysis. One and zero in the table present evaluations as 'yes (present)' and 'no (absent)', respectively. Total score was 11 for the highest quality paper. Study quality was considered higher, if the score gets close to 11.

References

1. Bock, B. C.; Thind, H.; Fava, J. L.; Dunsiger, S.; Guthrie, K. M.; Stroud, L.; Gopalakrishnan, G.; Sillice, M.; Wu, W., Feasibility of yoga as a complementary therapy for patients with type 2 diabetes: The Healthy Active and in Control (HA1C) study. *Complement Ther Med* **2019,** 42, 125-131.
2. Li, X.; Si, H.; Chen, Y.; Li, S.; Yin, N.; Wang, Z., Effects of fitness qigong and tai chi on middle-aged and elderly patients with type 2 diabetes mellitus. *PLoS One* **2020,** 15, (12), e0243989.
3. Ellis, D. A.; Carcone, A. I.; Slatcher, R.; Naar-King, S.; Hains, A.; Graham, A.; Sibinga, E., Efficacy of mindfulness-based stress reduction in emerging adults with poorly controlled, type 1 diabetes: A pilot randomized controlled trial. *Pediatr Diabetes* **2019,** 20, (2), 226-234.
4. Chan, A. W. K.; Chair, S. Y.; Lee, D. T. F.; Leung, D. Y. P.; Sit, J. W. H.; Cheng, H. Y.; Taylor-Piliae, R. E., Tai Chi exercise is more effective than brisk walking in reducing cardiovascular disease risk factors among adults with hypertension: A randomised controlled trial. *Int J Nurs Stud* **2018,** 88, 44-52.
5. Yadav, R.; Yadav, R. K.; Khadgawat, R.; Pandey, R. M., Comparative efficacy of a 12 week yoga-based lifestyle intervention and dietary intervention on adipokines, inflammation, and oxidative stress in adults with metabolic syndrome: a randomized controlled trial. *Transl Behav Med* **2019,** 9, (4), 594-604.
6. Leung, L. Y.; Chan, A. W.; Sit, J. W.; Liu, T.; Taylor-Piliae, R. E., Tai Chi in Chinese adults with metabolic syndrome: A pilot randomized controlled trial. *Complement Ther Med* **2019,** 46, 54-61.
7. Singh, A. K.; Kaur, N.; Kaushal, S.; Tyagi, R.; Mathur, D.; Sivapuram, M. S.; Metri, K.; Bammidi, S.; Podder, V.; Modgil, S.; Khosla, R.; Sharma, K.; Anand, A.; Malik, N.; Boroiah, V.; Nagarathna, R.; Nagendra, H. R.; Anand, A., Partitioning of radiological, stress and biochemical changes in pre-diabetic women subjected to Diabetic Yoga Protocol. *Diabetes Metab Syndr* **2019,** 13, (4), 2705-2713.
8. Raja-Khan, N.; Agito, K.; Shah, J.; Stetter, C. M.; Gustafson, T. S.; Socolow, H.; Kunselman, A. R.; Reibel, D. K.; Legro, R. S., Mindfulness-Based Stress Reduction in Women with Overweight or Obesity: A Randomized Clinical Trial. *Obesity (Silver Spring)* **2017,** 25, (8), 1349-1359.

9. Shomaker, L. B.; Bruggink, S.; Pivarunas, B.; Skoranski, A.; Foss, J.; Chaffin, E.; Dalager, S.; Annameier, S.; Quaglia, J.; Brown, K. W.; Broderick, P.; Bell, C., Pilot randomized controlled trial of a mindfulness-based group intervention in adolescent girls at risk for type 2 diabetes with depressive symptoms. *Complement Ther Med* **2017,** 32, 66-74.
10. Tillin, T.; Tuson, C.; Sowa, B.; Chattopadhyay, K.; Sattar, N.; Welsh, P.; Roberts, I.; Ebrahim, S.; Kinra, S.; Hughes, A.; Chaturvedi, N., Yoga and Cardiovascular Health Trial (YACHT): a UK-based randomised mechanistic study of a yoga intervention plus usual care versus usual care alone following an acute coronary event. *BMJ Open* **2019,** 9, (11), e030119.
11. Zheng, G.; Zheng, X.; Li, J.; Duan, T.; Ling, K.; Tao, J.; Chen, L., Effects of Tai Chi on Cerebral Hemodynamics and Health-Related Outcomes in Older Community Adults at Risk of Ischemic Stroke: A Randomized Controlled Trial. *J Aging Phys Act* **2019,** 27, (5), 678-687.
12. Patil, S. G.; Aithala, M. R.; Naregal, G. V.; Shanmukhe, A. G.; Chopade, S. S., Effect of yoga on cardiac autonomic dysfunction and insulin resistance in non-diabetic offspring of type-2-diabetes parents: A randomized controlled study. *Complement Ther Clin Pract* **2019,** 34, 288-293.
13. Chen, S. M.; Lin, H. S.; Atherton, J. J.; MacIsaac, R. J.; Wu, C. J., Effect of a mindfulness programme for long-term care residents with type 2 diabetes: A cluster randomised controlled trial measuring outcomes of glycaemic control, relocation stress and depression. *Int J Older People Nurs* **2020,** 15, (3), e12312.
14. Nijjar, P. S.; Connett, J. E.; Lindquist, R.; Brown, R.; Burt, M.; Pergolski, A.; Wolfe, A.; Balaji, P.; Chandiramani, N.; Yu, X.; Kreitzer, M. J.; Everson-Rose, S. A., Randomized Trial of Mindfulness-Based Stress Reduction in Cardiac Patients Eligible for Cardiac Rehabilitation. *Sci Rep* **2019,** 9, (1), 18415.
15. Meyer, J. D.; Hayney, M. S.; Coe, C. L.; Ninos, C. L.; Barrett, B. P., Differential Reduction of IP-10 and C-Reactive Protein via Aerobic Exercise or Mindfulness-Based Stress-Reduction Training in a Large Randomized Controlled Trial. *J Sport Exerc Psychol* **2019,** 41, (2), 96-106.
16. Qi, D.; Wong, N. M. L.; Shao, R.; Man, I. S. C.; Wong, C. H. Y.; Yuen, L. P.; Chan, C. C. H.; Lee, T. M. C., Qigong exercise enhances cognitive functions in the elderly via an interleukin-6-hippocampus pathway: A randomized active-controlled trial. *Brain Behav Immun* **2021,** 95, 381-390.
17. Puhlmann, L. M. C.; Engert, V.; Apostolakou, F.; Papassotiriou, I.; Chrousos, G. P.; Vrticka, P.; Singer, T., Only vulnerable adults show change in chronic low-grade inflammation after contemplative mental training: evidence from a randomized clinical trial. *Sci Rep* **2019,** 9, (1), 19323.
18. Vera, F. M.; Manzaneque, J. M.; Rodriguez, F. M.; Bendayan, R.; Fernandez, N.; Alonso, A., Acute Effects on the Counts of Innate and Adaptive Immune Response Cells After 1 Month of Taoist Qigong Practice. *Int J Behav Med* **2016,** 23, (2), 198-203.
19. Turner, L.; Galante, J.; Vainre, M.; Stochl, J.; Dufour, G.; Jones, P. B., Immune dysregulation among students exposed to exam stress and its mitigation by mindfulness training: findings from an exploratory randomised trial. *Sci Rep* **2020,** 10, (1), 5812.

20. Witek Janusek, L.; Tell, D.; Mathews, H. L., Mindfulness based stress reduction provides psychological benefit and restores immune function of women newly diagnosed with breast cancer: A randomized trial with active control. *Brain Behav Immun* **2019,** 80, 358-373.
21. Kenne Sarenmalm, E.; Martensson, L. B.; Andersson, B. A.; Karlsson, P.; Bergh, I., Mindfulness and its efficacy for psychological and biological responses in women with breast cancer. *Cancer Med* **2017,** 6, (5), 1108-1122.
22. Sohl, S. J.; Tooze, J. A.; Johnson, E. N.; Ridner, S. H.; Rothman, R. L.; Lima, C. R.; Ansley, K. C.; Wheeler, A.; Nicklas, B.; Avis, N. E.; Wagner, L. I., A Randomized Controlled Pilot Study of Yoga Skills Training Versus an Attention Control Delivered During Chemotherapy Administration. *J Pain Symptom Manage* **2022,** 63, (1), 23-32.
23. Huberty, J.; Eckert, R.; Dueck, A.; Kosiorek, H.; Larkey, L.; Gowin, K.; Mesa, R., Online yoga in myeloproliferative neoplasm patients: results of a randomized pilot trial to inform future research. *BMC Complement Altern Med* **2019,** 19, (1), 121.
24. Gautam, S.; Tolahunase, M.; Kumar, U.; Dada, R., Impact of yoga based mind-body intervention on systemic inflammatory markers and co-morbid depression in active Rheumatoid arthritis patients: A randomized controlled trial. *Restor Neurol Neurosci* **2019,** 37, (1), 41-59.
25. Ganesan, S.; Gaur, G. S.; Negi, V. S.; Sharma, V. K.; Pal, G. K., Effect of Yoga Therapy on Disease Activity, Inflammatory Markers, and Heart Rate Variability in Patients with Rheumatoid Arthritis. *J Altern Complement Med* **2020,** 26, (6), 501-507.
26. Mao, S.; Liang, Y.; Chen, P.; Zhang, Y.; Yin, X.; Zhang, M., In-depth proteomics approach reveals novel biomarkers of cardiac remodelling after myocardial infarction: An exploratory analysis. *J Cell Mol Med* **2020,** 24, (17), 10042-10051.
27. Marciniak, R.; Sumec, R.; Vyhnalek, M.; Bendickova, K.; Laznickova, P.; Forte, G.; Jelenik, A.; Rimalova, V.; Fric, J.; Hort, J.; Sheardova, K., The Effect of Mindfulness-Based Stress Reduction (MBSR) on Depression, Cognition, and Immunity in Mild Cognitive Impairment: A Pilot Feasibility Study. *Clin Interv Aging* **2020,** 15, 1365-1381.
28. Ng, T. K. S.; Fam, J.; Feng, L.; Cheah, I. K.; Tan, C. T.; Nur, F.; Wee, S. T.; Goh, L. G.; Chow, W. L.; Ho, R. C.; Kua, E. H.; Larbi, A.; Mahendran, R., Mindfulness improves inflammatory biomarker levels in older adults with mild cognitive impairment: a randomized controlled trial. *Transl Psychiatry* **2020,** 10, (1), 21.
29. Nugent, N. R.; Brick, L.; Armey, M. F.; Tyrka, A. R.; Ridout, K. K.; Uebelacker, L. A., Benefits of Yoga on IL-6: Findings from a Randomized Controlled Trial of Yoga for Depression. *Behav Med* **2021,** 47, (1), 21-30.
30. Andres-Rodriguez, L.; Borras, X.; Feliu-Soler, A.; Perez-Aranda, A.; Rozadilla-Sacanell, A.; Montero-Marin, J.; Maes, M.; Luciano, J. V., Immune-inflammatory pathways and clinical changes in fibromyalgia patients treated with Mindfulness-Based Stress Reduction (MBSR): A randomized, controlled clinical trial. *Brain Behav Immun* **2019,** 80, 109-119.

31. Buijze, G. A.; De Jong, H. M. Y.; Kox, M.; van de Sande, M. G.; Van Schaardenburg, D.; Van Vugt, R. M.; Popa, C. D.; Pickkers, P.; Baeten, D. L. P., An add-on training program involving breathing exercises, cold exposure, and meditation attenuates inflammation and disease activity in axial spondyloarthritis - A proof of concept trial. *PLoS One* **2019,** 14, (12), e0225749.
32. Cohen, Z. P.; Cosgrove, K. T.; Akeman, E.; Coffey, S.; Teague, K.; Hays-Grudo, J.; Paulus, M. P.; Aupperle, R. L.; Kirlic, N., The effect of a mindfulness-based stress intervention on neurobiological and symptom measures in adolescents with early life stress: a randomized feasibility study. *BMC Complement Med Ther* **2021,** 21, (1), 123.
33. Dada, T.; Mittal, D.; Mohanty, K.; Faiq, M. A.; Bhat, M. A.; Yadav, R. K.; Sihota, R.; Sidhu, T.; Velpandian, T.; Kalaivani, M.; Pandey, R. M.; Gao, Y.; Sabel, B. A.; Dada, R., Mindfulness Meditation Reduces Intraocular Pressure, Lowers Stress Biomarkers and Modulates Gene Expression in Glaucoma: A Randomized Controlled Trial. *J Glaucoma* **2018,** 27, (12), 1061-1067.
34. Hecht, F. M.; Moskowitz, J. T.; Moran, P.; Epel, E. S.; Bacchetti, P.; Acree, M.; Kemeny, M. E.; Mendes, W. B.; Duncan, L. G.; Weng, H.; Levy, J. A.; Deeks, S. G.; Folkman, S., A randomized, controlled trial of mindfulness-based stress reduction in HIV infection. *Brain Behav Immun* **2018,** 73, 331-339.
35. Hoge, E. A.; Bui, E.; Palitz, S. A.; Schwarz, N. R.; Owens, M. E.; Johnston, J. M.; Pollack, M. H.; Simon, N. M., The effect of mindfulness meditation training on biological acute stress responses in generalized anxiety disorder. *Psychiatry Res* **2018,** 262, 328-332.
36. Lengacher, C. A.; Reich, R. R.; Paterson, C. L.; Shelton, M.; Shivers, S.; Ramesar, S.; Pleasant, M. L.; Budhrani-Shani, P.; Groer, M.; Post-White, J.; Johnson-Mallard, V.; Kane, B.; Cousin, L.; Moscoso, M. S.; Romershausen, T. A.; Park, J. Y., A Large Randomized Trial: Effects of Mindfulness-Based Stress Reduction (MBSR) for Breast Cancer (BC) Survivors on Salivary Cortisol and IL-6. *Biol Res Nurs* **2019,** 21, (1), 39-49.
37. Li, G.; Huang, P.; Cui, S. S.; Tan, Y. Y.; He, Y. C.; Shen, X.; Jiang, Q. Y.; Huang, P.; He, G. Y.; Li, B. Y.; Li, Y. X.; Xu, J.; Wang, Z.; Chen, S. D., Mechanisms of motor symptom improvement by long-term Tai Chi training in Parkinson's disease patients. *Transl Neurodegener* **2022,** 11, (1), 6.
38. Meesters, A.; den Bosch-Meevissen, Y.; Weijzen, C. A. H.; Buurman, W. A.; Losen, M.; Schepers, J.; Thissen, M.; Alberts, H.; Schalkwijk, C. G.; Peters, M. L., The effect of Mindfulness-Based Stress Reduction on wound healing: a preliminary study. *J Behav Med* **2018,** 41, (3), 385-397.
39. Mirmahmoodi, M.; Mangalian, P.; Ahmadi, A.; Dehghan, M., The Effect of Mindfulness-Based Stress Reduction Group Counseling on Psychological and Inflammatory Responses of the Women With Breast Cancer. *Integr Cancer Ther* **2020,** 19, 1534735420946819.
40. Sungkarat, S.; Boripuntakul, S.; Kumfu, S.; Lord, S. R.; Chattipakorn, N., Tai Chi Improves Cognition and Plasma BDNF in Older Adults With Mild Cognitive Impairment: A Randomized Controlled Trial. *Neurorehabil Neural Repair* **2018,** 32, (2), 142-149.

41. Villalba, D. K.; Lindsay, E. K.; Marsland, A. L.; Greco, C. M.; Young, S.; Brown, K. W.; Smyth, J. M.; Walsh, C. P.; Gray, K.; Chin, B.; Creswell, J. D., Mindfulness training and systemic low-grade inflammation in stressed community adults: Evidence from two randomized controlled trials. *PLoS One* **2019,** 14, (7), e0219120.
